# Effects of nocturnal melatonin-based tissue-bone homeostasis manipulation at varying time points on pain and central mechanisms in individuals with knee osteoarthritis: a randomized controlled trial

**DOI:** 10.3389/fbioe.2026.1706840

**Published:** 2026-04-10

**Authors:** Liming Jiang, Yifan Wang, Zheng Xu, Yuwu Ding, Tianzhong Zi, Jiaxuan Bian, Kun Yang, Jiayi Xia, Yuelong Cao

**Affiliations:** 1 Department of Rehabilitation, Seventh People’s Hospital of Shanghai University of Traditional Chinese Medicine, Shanghai, China; 2 Institute of Specialized Diagnosis and Treatment Technology, Shuguang Hospital, Shanghai University of Traditional Chinese Medicine, Shanghai, China; 3 School of Rehabilitation Medicine, Shanghai University of Traditional Chinese Medicine, Shanghai, China; 4 Medical Laboratory Department, Seventh People’s Hospital of Shanghai University of Traditional Chinese Medicine, Shanghai, China; 5 Clinical Research Centre, The Second Rehabilitation Hospital of Shanghai, Shanghai, China

**Keywords:** central mechanism, knee osteoarthritis, nocturnal melatonin, pain, tissue-bone homeostasis manipulation

## Abstract

**Background:**

Tissue-bone homeostasis manipulation (TBHM) has been proven effective for knee osteoarthritis (KOA), but its optimal timing and underlying mechanisms remain unclear. Melatonin serves as a key biomarker of circadian rhythm, while electroencephalography (EEG) evaluates pain-related central mechanisms. This study investigated the efficacy of TBHM at different time points based on circadian principles and explored potential central mechanisms using EEG.

**Methods:**

In this 4-week randomized controlled single-blind trial, 88 KOA patients were randomized into four groups: group A (TBHM at 8 a.m.), group B (TBHM at 1 p.m.), group C (TBHM at 6 p.m.), and group D (joint mobilization). Interventions were administered once daily (20 min/session, 5 days/week). Primary outcome was Visual Analog Scale (VAS) for pain; secondary outcomes were resting-state EEG and Hamilton Anxiety Rating Scale. Salivary melatonin levels were measured to explore circadian mechanisms. Assessments were conducted at baseline and after 4 weeks. Statistical analyses employed two-way repeated-measures ANOVA.

**Results:**

Of the 88 patients randomized, 82 completed the study. After 4 weeks, all groups showed reduced VAS scores and increased melatonin levels. Post-treatment, group C exhibited significantly lower VAS scores than group B (FDR adjusted *P* = 0.023), and group B had significantly lower VAS scores than group A (FDR adjusted *P* = 0.037). Although group A showed lower scores than group D, the difference was not statistically significant (FDR adjusted *P* > 0.05). Melatonin levels increased significantly in the three TBHM groups after treatment. group C was demonstrated significantly higher melatonin levels than group B (FDR adjusted *P* < 0.001), group B was significantly higher than group A (FDR adjusted *P* = 0.007), and group A was higher than group D (FDR adjusted *P* = 0.026). After treatment, a decrease in β band activity and an increase in θ band activity were observed in the frontal and central regions of groups C and B in EEG analysis, but there was no significant difference (FDR adjusted *P* > 0.05).

**Conclusion:**

The TBHM at all time points can better improve the pain of KOA patients than joint mobilization, regulate cortical electrical activity, and increase the secretion of melatonin at night.

**Trial Registration:**

ChiCTR2400080820. Registered on Feb.07,2024.

## 1 Introduction

Knee osteoarthritis (KOA) is a prevalent degenerative joint disorder characterized by the deterioration of joint structures, which results in pain, stiffness, and functional impairments (Belluzzi et al., 2017; Battistelli et al., 2019). The primary risk factors associated with KOA include age, gender, and body mass index (BMI) (Michael, 2001; Li M. et al., 2025). A study shows that the prevalence of KOA increases with age, with a significantly higher incidence in people aged 70 and older (Kurniari et al., 2025). Furthermore, epidemiological studies suggest that women are at a greater risk of developing KOA compared to men, and an elevated BMI is correlated with an increased likelihood of disease onset (Warasna et al., 2025). KOA has been listed as a main cause of functional deficits in adults aged 60 and over (Collaborators, 2021).

Pain is the predominant symptom experienced by patients with KOA, typically presenting with less intensity during daylight hours and increasing in severity at night (Sasaki et al., 2014). This pain not only imposes physical strain but also significantly disrupts sleep quality (Parmelee et al., 2015). Such sleep disturbances, in turn, exacerbate pain, creating a self-perpetuating cycle (Sasaki et al., 2014). Numerous studies have demonstrated that enhancing sleep quality can substantially alleviate KOA-related pain. This relationship is largely attributed to the fact that sleep disturbances augment inflammatory processes, thereby intensifying pain symptoms in individuals with KOA (Irwin et al., 2016). Adequate sleep may contribute to the regulation of these inflammatory mechanisms, thus mitigating pain (Irwin et al., 2016). Consequently, a more comprehensive knowing of the pathophysiology of sleep disorders could lead to more effective treatments for KOA pain. Researches indicated that sleep disturbances were intricately linked to disruptions in the circadian rhythm (Yaron, 2003; Jiao et al., 2025). The natural internal process of regulating the sleep-wake cycle is repeated approximately every 24 h (Scott S et al., 2004; Martinez-Moral and Kannan, 2025). Retinal light signals regulate the circadian rhythm via the hypothalamus’s suprachiasmatic nucleus (SCN), syncing it with the rotation of the Earth (Bhaskar et al., 2025). Furthermore, much evidence indicates that circadian disruption exacerbates pain in various chronic conditions, including knee osteoarthritis (Bellamy et al., 1990; Guo et al., 2015). Conversely, restoration of circadian rhythmicity may alleviate pain. Mechanistically, circadian rhythms regulate inflammatory cytokines, pain-related neurotransmitters, and endogenous analgesic pathways (Du et al., 2022). Melatonin, a hormone from the pineal gland of the brain (Steven M et al., 1988; Abdi et al., 2021), significantly impacts biological processes like gene expression, inflammation, and tissue repair (Du et al., 2022). Monitoring the levels and concentration curves of melatonin release provides an effective means of assessing the body’s circadian rhythm (Frank and Charles, 2005; Kennaway, 2020). Disruptions in the circadian rhythm can lead to abnormal nocturnal melatonin secretion, which adversely affects sleep quality and exacerbates pain symptoms. Consequently, changes in melatonin secretion may indirectly reflect pain modulation. Employing the principle of circadian rhythm to guide artificial interventions in the management of KOA holds significant potential (Frank and Charles, 2005; Li H. et al., 2025).

In addition, much emerging research evidence shows that chronic musculoskeletal pain, including KOA, is associated with maladaptive neuroplastic changes in the central nervous system (Iuamoto et al., 2022). Resting-state electroencephalography (EEG) has been increasingly used to investigate pain-related cortical oscillations in chronic pain conditions (Zebhauser et al., 2023). Recent studies have demonstrated that patients with KOA exhibit distinct EEG oscillatory patterns compared to healthy controls, particularly increased β power and reduced θ activity in frontocentral regions, which correlate with pain intensity and disease severity (Simis et al., 2022). These findings suggest that EEG oscillations may serve as potential biomarkers of central sensitization and maladaptive compensatory mechanisms in KOA. Therefore, incorporating EEG assessment in KOA intervention studies can provide valuable insights into the central mechanisms underlying treatment effects, beyond peripheral pain relief.

Currently, KOA pain is predominantly managed with pharmacological interventions, including nonsteroidal anti-inflammatory drugs (NSAIDs) and opioids (Shourabi et al., 2024). However, these medications are frequently associated with gastrointestinal adverse effects and carry a risk of dependence (Bjordal et al., 2004; Dowell et al., 2016; Zhang et al., 2019). Consequently, non-drug treatments are urgently needed to alleviate pain without incurring significant side effects. Among the non-pharmacological approaches for managing KOA pain, manual therapy (MT) is widely endorsed by patients due to its efficacy in pain relief and its favorable safety and comfort profile (Silvernail et al., 2024). Tissue-bone homeostasis manipulation (TBHM) is a prevalent form of MT, emphasizing the importance of both soft tissues (tendons, ligaments, muscles) and bones (Zhang et al., 2024). In traditional Chinese medicine, “homeostasis” describes the mechanical harmony between soft tissues and bones. Specifically, TBHM aims to restore the alignment of bones (correcting the force line) and the proper tension, elasticity, and relaxation of surrounding soft tissues. This mechanical balance is essential for joint function and pain relief in KOA. The final phase involves fully releasing the infrapatellar fat pad from its adhesions through a specialized release technique. Ultimately, realigning the displaced patella and tibiofemoral joint improves patellar movement and restores mechanical axis of the knee, leading to tendon relaxation, bone alignment, and pain relief (Zhang et al., 2024). Notably, our clinical observations indicate that the therapeutic effects of TBHM on KOA pain vary depending on the timing of application. This observation may align with the findings of Carlson et al., which suggest that varying exercise time points exert distinct therapeutic effects on patients with KOA (Carlson et al., 2019). This observation implies the potential for optimizing treatment timing in accordance with circadian rhythms. Despite the significant pain reduction associated with TBHM in KOA patients, the best time for its application is still unclear (Zhang et al., 2024).

In conclusion, this research sought to investigate the effects of TBHM at various time points on KOA, offering a novel perspective on nocturnal melatonin-based traditional manipulation therapy. Additionally, by using EEG to deeply study the mechanisms through which TBHM influences patients' physiological states, we aim to augment our understanding of pain management and physiological regulation in individuals with KOA, thereby establishing a robust theoretical foundation for future clinical practice.

## 2 Materials and methods

### 2.1 Study design

**FIGURE 1:**
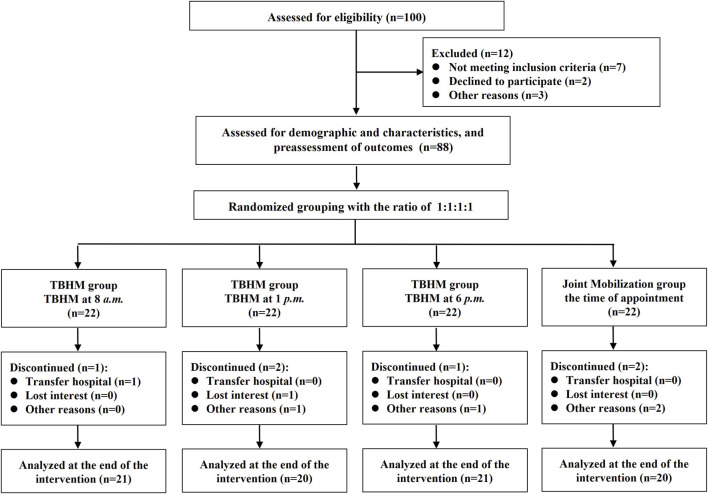
A concise flowchart of the patient recruitment and retention process. TBHM: Tissue-bone homeostasis manipulation.

This research recruited patients with KOA from Shanghai Seventh People’s Hospital and the surrounding community between July and September 2025. The patients’ recruitment and retention process were shown in Figure 1. The recruitment process was managed by a dedicated individual to ensure strict adherence to the protocol. In preparing this manuscript, we followed the Consolidated Standards of Reporting Trials statement (Hopewell et al., 2025). All patients provided written informed consent before enrollment.

### 2.2 Participants

#### 2.2.1 Inclusion criteria

1. Meets the diagnostic criteria for KOA established by the American College of Rheumatology in 1986;
2. Age 50–75 years;
3. Kellgren-Lawrence radiographic classification II-III (Kellgren and Lawrence, 1957; Altman, 1991);
4. Signed informed consent form.

#### 2.2.2 Exclusion criteria

1. Patients who have undergone knee surgery (Azizi et al., 2021; Xia et al., 2025);
2. Patients with knee infections;
3. Patients with mental illness;
4. Cancer patients;
5. Patients who have received knee treatment within the past 3 months;
6. Patients who cannot undergo electroencephalogram (EEG) assessment (scalp sensitivity, skin wounds, inability to cooperate).

#### 2.2.3 Drop-out criteria

1. Treatments were not completed as required.
2. Adverse events prevented the continuation of treatment.
3. Acceptance of other treatments that could affect study results.

### 2.3 Sample size calculation

The G*Power software (v3.1.9.2, University of Dusseldorf, Germany) was used for sample size calculation with two-way repeated measures analysis of variance (ANOVA). According to previous study (Cohen, 1992), the effect size η_p_^2^ = 0.06 (Cohen’s f = 0.26), alpha = 0.05, power (1-β = 0.95), repeated measures correlation ρ = 0.4, and considering sphericity correction (ε = 1), and the total sample size was 100 (including a 20% dropout rate).

### 2.4 Randomization, allocation concealment and blinding

An independent researcher, uninvolved with patients or data collection, used Microsoft Excel 2013 for computerized randomization. The 88 patients who met the inclusion criteria were randomized into three TBHM groups (A, B, C) at varying time points (8 a.m., 1 p.m., 6 p.m.) and a joint mobilization group (D). The entire randomization process was performed by an independent researcher who used sealed opaque envelopes to conceal the serial numbers and followed the established order to open each envelope sequentially only after each patient completed baseline testing to prevent selection bias. Due to the visual visibility of TBHM and joint mobilization treatments, both the interventionists and the patients were inevitably aware of the assigned intervention. To ensure objectivity and eliminate bias, assessors and statisticians were blinded to the intervention and not involved in patient recruitment, maintaining neutrality in data collection and analysis.

### 2.5 Interventions

All patients in this study were treated with either TBHM or joint mobilization. To ensure consistency of intervention, two physical therapists with more than 3 years of clinical experience and valid rehabilitation therapy certificates were selected to perform TBHM and joint mobilization interventions, respectively. All intervention participants completed relevant methodological training before the study began to ensure the accuracy of the intervention methods.

#### 2.5.1 TBHM

1) Muscle relaxation: The patient reclines as the therapist applies rolling, kneading, and pressing techniques on the thigh and calf muscles for 5 min 2) Acupoint pressing: Apply pressure to acupoints such as “Liangqiu,” “Xuehai,” “Neixiyan,” “Waixiyan,” “Yanglingquan,” “Zusanli,” and “Ashi” for 5 min, ensuring the pressure is comfortable and induces a “deqi” sensation. 3) Infrapatellar fat pad release: With the patient relaxed and lying back, the therapist bends the affected knee slightly, places thumbs on the patellar tendon, and fingers behind the knee. Subsequently, the therapist elevates the heel, applies pressure towards the femoral condyles, and extends the knee. 4) Patella Manipulation: The patients lie on their back and relax. The therapist stabilizes and gently moves the outer patella to its maximum range, holding briefly before returning, repeated three times. 5) Tibia Rotation: The patients lie with a slightly bent knee. The therapist positions the thigh at the edge of the bed, grips the knee, and rotates the tibia inward and outward to its limit, holding briefly, then relaxing, repeated three times.

Previous research has demonstrated that exercise timing could differentially affect melatonin secretion, with morning workouts boosting it and afternoon sessions reducing it (Carlson et al., 2019). In addition, to ensure patient compliance, the study scheduled 20-min TBHM sessions at 8 a.m., 1 p.m., and 6 p.m., once daily, 5 days a week for 4 weeks. Throughout the intervention, we maintained continuous communication with the patients so that the intensity of the technique could be adjusted in a timely manner.

#### 2.5.2 Joint mobilization

1) Long-axis Traction (Kaya Mutlu et al., 2018): The patients are positioned in a seated posture with the affected knee flexed, allowing the leg to dangle over the edge of the bed. The therapist pulls the ankle towards the foot for 15 s, repeating this 5 times. 2) Anterior-Posterior Glide: The patients sit with the affected leg bent. The therapist stabilizes the leg and pushes the upper tibia backward for 15 s, repeating this 5 times. 3) The patients are positioned in a supine posture with the affected hip and knee flexed. The therapist, positioned on the affected side, places their thumb on the proximal tibia and their fingers in the popliteal fossa, subsequently leaning back to apply an anterior force to the tibia. Each glide is maintained for a duration of 15 s and is repeated five times daily, 5 days per week, over a period of 4 weeks. The session lasts 20 min with force adjusted to patient tolerance (grade 3–4). The treatment time of the joint mobilization group varies by each patient’s appointment time. Joint mobilization was chosen as a standard intervention because it is a well-established approach in KOA rehabilitation. Unlike TBHM, which prioritizes muscle coordination over skeletal alignment, joint mobilization prioritizes skeletal alignment. Moreover, previous research has also demonstrated that the effectiveness of joint mobilization is consistent regardless of the timing of treatment (Yacks et al., 2025).

### 2.6 Outcome measurements

The assessments of this study were divided into three distinct components: Patients’ demographic and clinical characteristics were extensively examined, including age, sex, weight, height, body mass index (BMI), disease site, disease duration, and K-L grade; primary outcomes included changes in visual analog scale (VAS) pain scores and salivary melatonin levels; and secondary outcomes included resting electroencephalogram (EEG) changes and Hamilton Anxiety Rating Scale (HAMA) scores. Both primary and secondary outcome assessments were conducted before the start of the intervention and after the completion of the 4-week treatment period. To ensure the reliability and accuracy of the assessments and to avoid any potential discrepancies in the results, One physician with over 5 years of clinical rehabilitation assessment experience and a valid rehabilitation physician certificate was selected to perform all assessment tasks except for the salivary melatonin level test; and one laboratory technician with over 10 years of clinical laboratory experience and a valid clinical laboratory technician certificate was selected to perform the salivary melatonin level test. All evaluators also completed relevant methodological training before the study began to ensure the accuracy of the evaluation methods. Meanwhile, to ensure better consistency in the assessment, the same assessment task was performed by the same assessor for all patients before and after treatment. Furthermore, to maintain impartiality, data collection and analysis were conducted by individuals independent of the clinical research team.

#### 2.6.1 Primary outcomes

##### 2.6.1.1 VAS pain score

The VAS, a 10-cm line with 0 representing “no pain” and 10 representing “worst pain possible,” was used as the primary outcome measure to assess improvement 32. Patients were asked to mark the line based on the severity of their knee pain. The VAS has been proven reliable and valid for musculoskeletal conditions (Wewers and Lowe, 1990). To assess the clinical significance of variations in VAS scores, the minimal clinically important difference (MCID) was employed, with a threshold established at 1.16 according to existing research finding (Lai et al., 2024).

##### 2.6.1.2 Melatonin content in saliva

###### 2.6.1.2.1 Saliva sample collection

The procedures for collecting saliva samples were uniformly taught by professional laboratory physicians to researchers or their immediate family members. For inpatients, researchers collected samples; for outpatients, immediate family members assisted in sample collection. Patients were instructed to rinse their mouths thoroughly at night and maintain a normal sleep schedule (Mahon et al., 2014). The following morning, researchers or immediate family members woke the patients and collected samples in dim light (around 3:00 a.m., when salivary melatonin secretion peaks) (Mahon et al., 2014; Carlson et al., 2019). Approximately 10 mL of saliva was collected by passive instillation into a polypropylene conical tube. The tube was sealed in an opaque black plastic bag and immediately frozen at −20 °C. The samples were delivered frozen to the laboratory the next day and stored in a −80 °C freezer until analysis (Sung et al., 2021). All samples were labeled only with a number to prevent individual identification by the laboratory technician during analysis.

###### 2.6.1.2.2 Melatonin analysis

For analysis, saliva samples were warmed to room temperature and centrifuged at 3000 rpm for 20 min. The supernatant was then analyzed. Salivary melatonin levels were measured using a competitive enzyme-linked immunosorbent assay (ELISA) (Sung et al., 2021) according to the manufacturer’s instructions (Human Salivary Melatonin ELISA Kit, H256-one to two, Nanjing Jiancheng Bioengineering Institute). All samples were run in duplicate. Intra-assay (repeatability) and inter-assay (reproducibility) coefficients of variation were <10% and <12%, respectively. Limit of detection: 10–3000 ng/L.

#### 2.6.2 Secondary outcomes

##### 2.6.2.1 EEG

###### 2.6.2.1.1 EEG acquisition and preprocessing

The resting-state EEG was collected by the assessor in a quiet room using a 32-channel EEG signal acquisition system (Xi’an ZhenTec Intelligence Technology Co., Ltd., China). The electrodes of the EEG cap were placed according to the international 10/20 system, covering brain regions including the frontal, central, and parietal areas. According to the manufacturer’s specifications, CPz was designated as the reference electrode, and ground electrode was placed on the forehead at the AFz location. Patients were instructed to maintain a comfortable sitting position and avoid overt limb movements or verbal communication to minimize motion artifacts. Data were collected with eyes closed for approximately 10 min. Prior to the experiment, the sampling rate was set to 1,000 Hz, with a 1–100 Hz bandpass filter and a 50 Hz notch filter to mitigate powerline interference (Wang et al., 2023). Electrode impedance was adjusted to below 20 kΩ using saline-soaked Lingke cotton (Zhang et al., 2023). EEG data preprocessing was performed using the EEGLAB toolbox, including channel localization, data filtering, baseline correction, re-referencing, and independent component analysis (ICA). During preprocessing, a 1–100 Hz bandpass filter and a 49–51 Hz notch filter was applied. ICA was computed to remove artifacts caused by ocular movements and muscle activity, thereby enhancing the signal-to-noise ratio (SNR) of the EEG data (Xu R. et al., 2023). Finally, the entire dataset underwent visual inspection to ensure quality.

###### 2.6.2.1.2 EEG analysis

**FIGURE 2:**
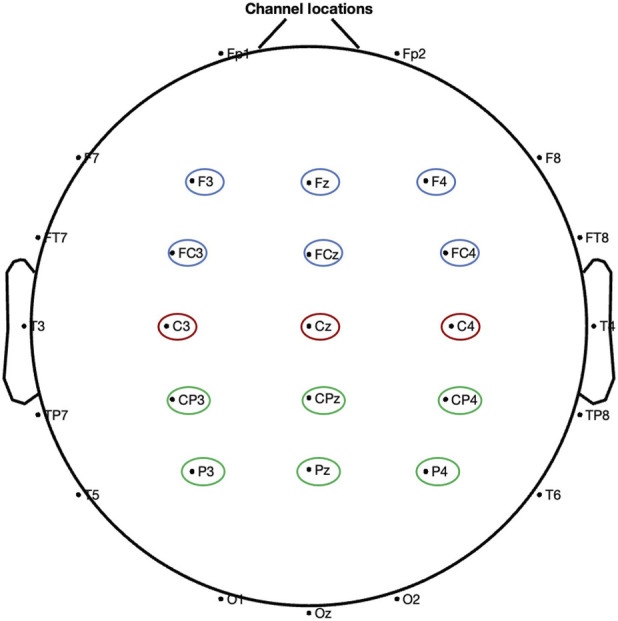
Electrode position assignment. The electrode with the blue circle represents the frontal, the electrode with the red circle represents the central region, and the electrode with the green circle represents the parietal.

EEG power spectral density (PSD) analysis. PSD analysis of EEG signals was conducted using a modified Welch periodogram method. The PSD for each channel was computed via the spectopo function embedded in EEGLAB. For group comparisons, PSD values were calculated at each frequency point from 1 to 50 Hz (in 0.5 Hz increments). At each frequency point, a one-way ANOVA was performed to compare PSD values among the four groups, and the resulting P-values were corrected using the False Discovery Rate (FDR) method. To assess PSD in data recorded from individual electrodes, PSD values were measured across baseline and after the end of the treatment within distinct frequency bands: δ (one to four Hz), θ (four to eight Hz), α (8–12 Hz), β (12–30 Hz), slow gamma (30–55 Hz), and fast gamma (55–100 Hz) (Wu et al., 2024). All measurements were calculated from three regions of interest (ROIs): frontal, central, and parietal regions, as these regions are closely associated with pain perception (Jensen et al., 2016). Electrodes representing these regions were selected and averaged; the electrode locations were shown in Figure 2.

##### 2.6.2.2 Hamilton Anxiety Rating Scale (HAMA)

The HAMA scale is crucial for evaluating anxiety severity. It consists of 14 items, each defined by a series of symptoms, and measures both mental anxiety (agitation and psychological distress) and somatic anxiety (physical symptoms related to anxiety) (Thompson, 2015). The study evaluated the impact of the TBHM treatment on anxiety by using HAMA to measure patients' anxiety levels before and after treatment (Xu H. et al., 2023).

### 2.7 Safety and adverse events

Adverse events are defined as any untoward medical occurrence that a patient experiences during the trial and may be related to the intervention. During this trial study, the safety of the intervention was assessed by evaluating, recording, and analyzing all adverse events that occurred, including the number of patients experiencing adverse events and their severity. Adverse events were classified as mild, moderate, or severe based on the type of adverse reaction and the duration of symptoms after treatment. According to previous literature (Tragord et al., 2013; Chan et al., 2023), adverse events associated with TBHM and joint mobilization interventions may include: local pain, redness and swelling at the treatment site, and discomfort within the joint. When these symptoms are relieved after a short rest, they are considered mild adverse reactions; when the symptoms persist for more than 24 h, they are considered moderate adverse reactions; and when the pain is unbearable and the discomfort within the joint leads to continued worsening of joint dysfunction, they are considered severe adverse reactions. Patients with moderate or severe adverse reactions are required to withdraw from the trial and receive timely symptomatic treatment to protect the rights of patients.

### 2.8 Statistical analysis

The results were evaluated through intention-to-treat (ITT) analysis, employing the last observation carried forward method to address missing data. Continuous variable data that conforms to normal distribution were reported by mean ± standard deviation or mean (95% CI), while nonnormal distribution data were reported by median and interquartile range. Categorical variables were expressed as frequencies. For continuous variables that satisfied the assumptions of normality and homogeneity of variance, a two-way repeated-measures ANOVA was applied; otherwise, the Wilcoxon test was employed. Categorical variables were assessed using the chi-square test. Furthermore, the correlations between absolute value of pain score difference and melatonin content difference were assessed using Pearson correlation coefficients or Spearman correlation coefficients (when normality was not met), which were categorized as low (r < 0.30), moderate (0.30 < r < 0.60), and high (r > 0.60) (Monticone et al., 2018). Results were deemed statistically significant with a p-value under 0.05. EEG data was preprocessed and analyzed with MATLAB 2022b. Data visualization was done using the EEGLAB toolbox for better understanding. IBM SPSS Statistics 25.0 (SPSS Inc., Chicago, IL, USA) was used for statistical analysis of all data.

## 3 Results

### 3.1 Participant baseline characteristics

Of the 100 cases assessed for eligibility, 88 participants who met the inclusion criteria completed the baseline characteristics assessment. Furthermore, in group A, one patient withdrew due to an acute attack of gastroenteritis. In group B, one patient withdrew due to a cold, while another withdrew due to distance caused by changing their place of residence. In group C, one patient withdrew due to inability to adhere to treatment. In group D, two patients withdrew due to inability to adhere to treatment. Finally, 82 cases completed the experiment and were included in the final analysis, including 21 cases in group A, 20 cases in group B, 21 cases in group C, and 20 cases in group D. Table 1 summarizes participants' baseline demographic and clinical characteristics. There were no significant differences in demographic and clinical characteristics among the four groups (FDR adjusted *P* > 0.05).

**TABLE 1:** Participant baseline characteristics.

Characteristics	Group A (n = 22)	Group B (n = 22)	Group C (n = 22)	Group D (n = 22)
Age (y)	63.59 ± 5.11	65.55 ± 5.36	64.18 ± 4.71	63.91 ± 3.62
Sex, female, n (%)	15 (68.18)	14 (63.63)	16 (72.73)	15 (68.18)
Height (cm)	163.20 ± 6.44	165.02 ± 7.55	163.57 ± 7.16	162.39 ± 7.48
Weight (kg)	71.49 ± 6.40	73.20 ± 7.92	70.42 ± 7.22	71.89 ± 9.01
BMI (kg/m^2^)	26.97 ± 3.58	26.95 ± 2.18	26.08 ± 2.86	27.29 ± 3.25
Injury side, right, n (%)	11 (50)	9 (40.91)	8 (36.36)	9 (40.91)
Time after onset (months)	9.14 ± 4.04	7.64 ± 2.74	8.00 ± 3.75	7.55 ± 2.76
K-L grade, II, n (%)	18 (81.82)	18 (81.82)	18 (81.82)	19 (86.36)

### 3.2 Primary outcome

#### 3.2.1 VAS pain score

Table 2 shows the score of the VAS between four groups at baseline and 4 weeks of treatment. The statistical results revealed a significant main effect of time (F = 387.882, *P* < 0.001) and a significant time × group interaction effect (F = 25.137, *P* < 0.001). Prior to the treatment, no significant differences were observed in VAS pain scores among the four groups (A, B, C, D) (FDR adjusted *P* > 0.05). Post-treatment, post-hoc tests demonstrated that compared to Group C, the VAS scores were significantly higher in Group B (FDR adjusted P = 0.023), Group A (FDR adjusted P < 0.001), and Group D (FDR adjusted P < 0.001), indicating that Group C achieved the greatest pain reduction. Additionally, compared to Group B, VAS scores were significantly higher in Group A (FDR adjusted P = 0.037) and Group D (FDR adjusted P = 0.029). Group A was smaller than group D, but there was no statistical difference (FDR adjusted *P* > 0.05). Within-group comparisons further confirmed significant decreases in VAS scores across all groups post-treatment compared to baseline (*P* < 0.001).

**TABLE 2:** Comparison of the score of the VAS between four groups at baseline and 4 weeks of treatment.

Test	Group	Baseline mean (95%CI)	4 Weeks mean (95%CI)	F	*P*	η^2^
VAS	Group A	4.48 (3.91, 5.05)	3.33 (2.79, 3.88)^ab^	40.121	<0.001	0.340
	Group B	4.65 (4.00, 5.30)	2.55 (2.04, 3.06)^a^	129.016	<0.001	0.623
	Group C	4.62 (4.03, 5.20)	1.67 (1.25, 2.08)	267.755	<0.001	0.774
	Group D	4.45 (3.65, 5.25)	3.45 (2.83, 4.07)^ab^	29.255	<0.001	0.273

^a^ *P* < 0.05 for comparison with group C, ^b^ *P* < 0.05 for comparison with group B.

#### 3.2.2 Melatonin content in saliva

Table 3 shows the score of the melatonin content between four groups at baseline and 4 weeks of treatment. The statistical results demonstrated significant main effects of time (F = 234.740, *P* < 0.001) and group (F = 13.877, *P* < 0.001), along with a significant time × group interaction (F = 44.316, *P* < 0.001). Baseline melatonin levels did not differ among groups A, B, C, and D (FDR adjusted *P* > 0.05). Post-treatment, post-hoc tests revealed statistically significant differences between all groups. Compared to Group C, melatonin levels were significantly lower in Group B (FDR adjusted P < 0.001), Group A (FDR adjusted P < 0.001), and Group D (FDR adjusted P < 0.001). Compared to Group B, melatonin levels were significantly lower in Group A (FDR adjusted P = 0.007) and Group D (FDR adjusted P < 0.001). Compared to Group A, melatonin levels were significantly lower in Group D (FDR adjusted P = 0.026). These results indicate that Group C achieved the most substantial increase in melatonin secretion, followed by Group B, then Group A, with Group D showing the smallest increase. Within-group comparisons confirmed significant post-treatment increases in melatonin levels for groups A, B, and C (*P* < 0.05). However, group D showed no significant difference from baseline to post-treatment (*P* > 0.05).

**TABLE 3:** Comparison of the melatonin content between four groups at baseline and 4 weeks of treatment.

Test	Group	Baseline mean (95%CI)	4 Weeks mean (95%CI)	F	*P*	η^2^
Melatonin content	Group A	14.28 (13.11, 15.46)	16.99 (15.50, 18.48)^ab^	20.173	<0.001	0.205
	Group B	14.31 (13.06, 15.55)	19.94 (18.39, 21.49)^a^	82.871	<0.001	0.515
	Group C	14.75 (13.17, 16.33)	24.63 (22.80, 26.47)	268.099	<0.001	0.775
	Group D	14.13 (12.90, 15.35)	14.62 (13.42, 15.82)^abc^	0.656	0.421	0.008

^a^ *P* < 0.05 for comparison with group C, ^b^ *P* <0.05 for comparison with group B, ^c^ *P*<0.05 for comparison with group A.

#### 3.2.3 Correlation between absolute value of pain score difference and melatonin content difference (post-treatment-pre-treatment)

**FIGURE 3:**
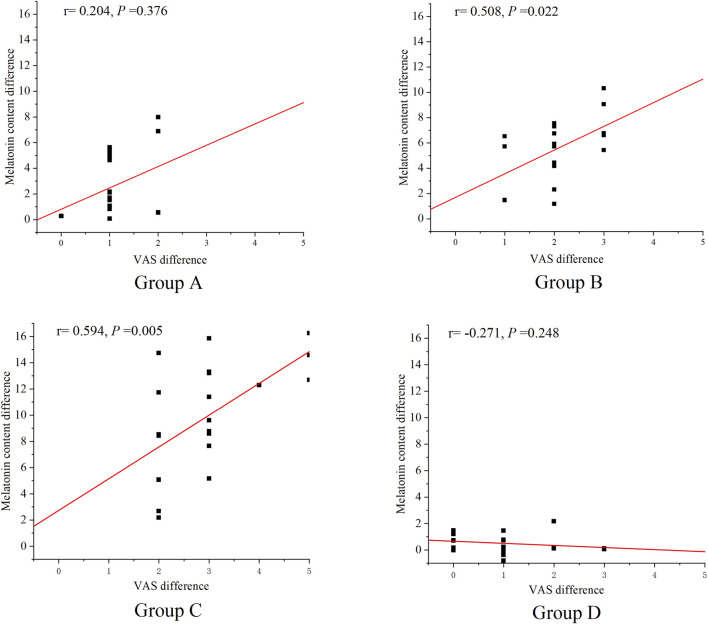
Correlation between absolute value of pain score difference and melatonin content difference.

According to Tables 2, 3, the pain scores of the four groups of KOA patients decreased and the melatonin content increased after treatment. Correlation analysis showed that the absolute value of the difference in VAS score in group B and group C was significantly positively correlated with the difference in melatonin content (Group B: moderate correlation: r = 0.508, *P* = 0.022; group C: moderate correlation: r = 0.594, *P* = 0.005). There was no significant correlation between the absolute value of VAS score difference and the difference in melatonin content in group A and group D (*P* > 0.05). As shown in Figure 3.

### 3.3 Secondary outcome

#### 3.3.1 Electroencephalogram (EEG)

**FIGURE 4:**
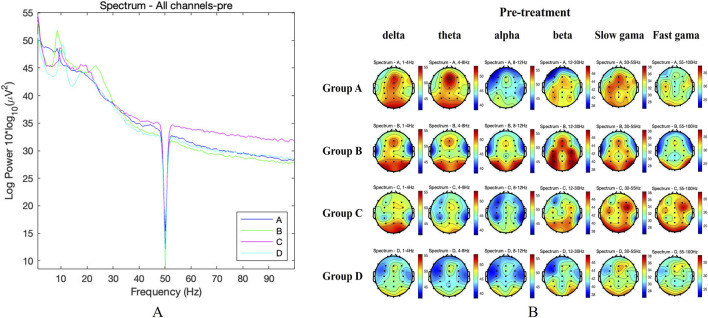
Global measures of brain activity before treatment. **(A)** Comparison of global power spectra of patients in the four groups before treatment. **(B)** Comparison of topographic maps of patients in the four groups before treatment.

**FIGURE 5:**
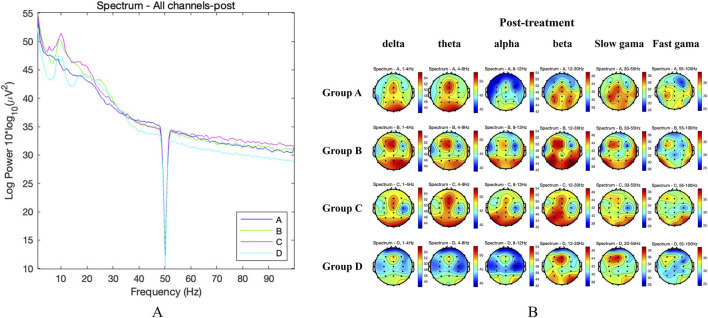
Global measures of brain activity after treatment. **(A)** Comparison of global power spectra of patients in the four groups after treatment. **(B)** Comparison of topographic maps of patients in the four groups after treatment.

For the global measures of brain activity, the global power spectra of the average EEG activity across all electrodes in the four groups of KOA patients were compared before treatment. The results showed no significant difference among the four groups at any frequency (FDR adjusted *P* > 0.05). The global power spectrum was shown in Figure 4A, and the topographic maps were shown in Figure 4B. After 4 weeks treatment, the above differences among the four groups of KOA patients were still not significant (FDR adjusted *P* > 0.05). The global power spectrum was shown in Figure 5A, and the topographic maps were shown in Figure 5B.

**FIGURE 6:**
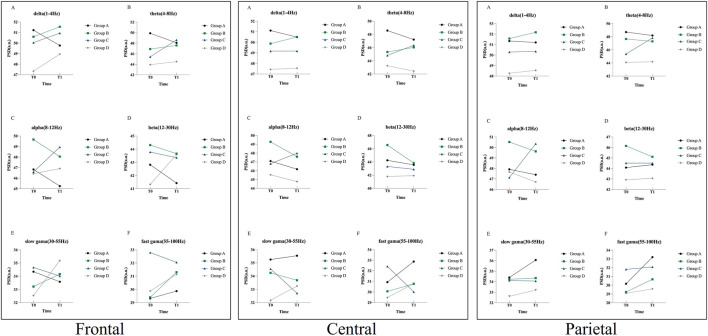
Differences in frontal, central and parietal PSD values among the four groups before and after treatment.

For the ROIs analysis, before treatment, there were no statistically significant differences in the PSD values of the four groups in each frequency band in the frontal, central and parietal areas (FDR adjusted *P* > 0.05). After 4 weeks of treatment, the above differences between the four groups in the frontal, central and parietal areas were still not significant (FDR adjusted *P* > 0.05). However, we found that the β-band activity in the frontal and central regions of Group C and B was weakened, while the θ-band activity was enhanced compared with before treatment. These characteristics were not observed in the other two groups, as demonstrated in Figure 6.

#### 3.3.2 Hamilton Anxiety Rating Scale (HAMA)

Table 4 shows the score of the HAMA values between four groups at baseline and 4 weeks of treatment. The statistical results revealed a significant main effect of time (F = 166.824, *P* < 0.001) and a significant time × group interaction effect (F = 16.938, *P* < 0.001). Baseline HAMA scores did not differ significantly among the four groups (A, B, C, D) (FDR adjusted *P* > 0.05). Post-treatment analyses showed that compared to Group C, HAMA scores were significantly higher in Group B (FDR adjusted P = 0.034), Group A (FDR adjusted P = 0.018), and Group D (FDR adjusted P = 0.022). However, there was no statistically significant difference among group B, A, and D (FDR adjusted *P* > 0.05). Notably, within-group comparisons demonstrated significant reductions in HAMA scores from baseline to post-treatment for all groups (*P* < 0.05).

**TABLE 4:** Comparison of the score of the HAMA between four groups at baseline and 4 weeks of treatment.

Test	Group	Baseline mean (95%CI)	4 Weeks mean (95%CI)	F	*P*	η^2^
HAMA	Group A	14.71 (13.02, 16.41)	13.24 (11.48, 15.00)^a^	14.514	<0.001	0.157
	Group B	15.40 (13.87, 16.93)	12.90 (11.64, 14.16)^a^	39.645	<0.001	0.337
	Group C	15.29 (13.71, 16.86)	10.48 (9.22, 11.74)	84.954	<0.001	0.664
	Group D	14.80 (13.29, 16.31)	13.45 (11.94, 15.96)^a^	11.561	0.001	0.129

^a^ *P* < 0.05 for comparison with group C.

### 3.4 Safety

During the study, one adverse reaction occurred in group A and one in group D. The former presented as transient localized knee pain after treatment, which gradually subsided after a short rest; the latter presented as transient joint discomfort, which also subsided after rest. No adverse reactions occurred in the other two groups. Overall, all four groups demonstrated a good safety profile, with no significant differences in the incidence of adverse events (*P* > 0.05).

## 4 Discussion

This study aimed to investigate the efficacy of TBHM at different time points for patients with KOA. Joint mobilization served as the control group. VAS pain scores and salivary melatonin levels were used to elucidate the optimal timing of TBHM and to reveal the relationship between efficacy and circadian rhythms. Furthermore, EEG was used to investigate the brain mechanisms underlying TBHM treatment for KOA. Results showed that after 4 weeks of treatment, VAS pain scores decreased and melatonin levels increased in all groups. Pain improvement was significantly better in the 6 p.m. TBHM group than in the 1 p.m. TBHM group; the 1 p.m. TBHM group was significantly better than the 8 a.m. TBHM group. While the 8 a.m. TBHM group showed better improvement compared to the joint mobilization group, the difference was not significant. Melatonin levels were significantly higher in the 6 p.m. TBHM group than in the 1 p.m. TBHM group, the 1 p.m. TBHM group was significantly higher than the 8 a.m. TBHM group, and the 8 a.m. TBHM group was significantly higher than the joint mobilization group. In terms of EEG analysis, there were no significant differences in global power spectral density among the four groups after treatment, but the 6 p.m. TBHM group and the 1 p.m. TBHM group showed decreased β band activity and increased θ band activity in the frontal and central regions.

Pain is a common symptom of KOA and significantly impacts patients' quality of life (Glyn-Jones et al., 2015; Chen et al., 2017). A study by Chan et al. showed that manipulation could relieve pain, improve stiffness, and enhance quality of life in KOA patients (Chan et al., 2023). A study by Feng et al. showed that manipulation therapy significantly could improve pain, disability, and functional activity in KOA patients (Feng et al., 2023). Our previous research also found that TBHM significantly improved pain, function, and gait in KOA patients (Zhang et al., 2024). This finding aligns with the results of the study. This study showed that TBHM at all three time points improved pain in KOA patients, with evening intervention being more effective than midday, and midday being more effective than morning. This may be related to the different effects of TBHM intervention at varying time points on nocturnal salivary melatonin secretion levels in KOA patients. Pain associated with KOA is affected by sleep patterns and circadian rhythms (Crodelle et al., 2023). Salivary melatonin, a hormone secreted by the pineal gland, plays an important role in regulating the sleep-wake cycle (Fowler et al., 2022). Its levels fluctuate closely with the body’s biological clock (Pundir et al., 2023). Its secretion exhibits a distinct circadian rhythm, with significantly higher levels at night than during the day. When circadian rhythms are disrupted, nocturnal salivary melatonin levels decrease (Carpi et al., 2024), and KOA patients experience more pronounced pain symptoms. Conversely, the greater the increase in nocturnal melatonin secretion, the more pronounced the pain improvement in KOA patients. The study found that evening interventions led to the highest nocturnal salivary melatonin release, followed by midday, and then morning interventions. This is consistent with the improvement in pain seen in KOA patients at different TBHM intervention times. Correlation analysis between pre- and post-treatment changes in salivary melatonin levels and the degree of pain improvement showed a positive correlation, further explaining why evening interventions are most effective in improving KOA pain.

In addition, we also observed the effects of TBHM treatment at different time points on cortical activity in KOA patients, using EEG to analyze the possible central mechanisms by which manipulation improves pain. KOA pain has traditionally been attributed to degeneration or damage to periarticular structures. However, the nature and intensity of pain often differ significantly from the severity of knee joint radiographs (Perrot et al., 2019). Some scholars have speculated that this may be related to altered cortical activity in patients with KOA (Finan et al., 2013; Mathew et al., 2024). Jerin et al. showed that the overall resting-state EEG signature of chronic pain in patients with KOA was characterized by increased β activity (Mathew et al., 2024). Gram et al., in a pain evoked and cortical response test in patients with hip arthritis, found that when pain was evoked, β activity in the EEG increased, while θ activity decreased (Gram et al., 2017). In a study by Simis et al. on pain and resting-state EEG in KOA patients, it was found that those with greater pain and degeneration showed increased frontocentral β activity and decreased θ activity (Simis et al., 2022). In a study of resting-state EEG in chronic pain patients, Dinh et al. found no differences in global and regional power spectral density measures of brain activity between chronic pain patients and healthy controls (Ta Dinh et al., 2019). These findings generally align with the results of the study. Our results also showed no significant differences in global and regional power spectral density measures in resting-state EEG in patients with KOA following TBHM intervention at different time points. However, β activity was reduced, while θ activity was enhanced, in the frontal and central regions of the 6 p.m. and 1 p.m. intervention groups. This may be because, while TBHM intervention in KOA does not alter global cortical properties, it does have a certain effect on patients' pain control and cortical-subcortical pain regulation. This is also reflected in the HAMA anxiety scores before and after treatment. Our results showed that the anxiety scores in the 6 p.m. intervention group were significantly lower than those in the other groups after treatment. This may be because the TBHM intervention at this time point showed the most significant increase in θ activity in the frontal and central regions. The θ band is known to be closely associated with emotional control and is a modulator of the affective network of pain control (Simis et al., 2022). Studies have shown that higher θ activity is associated with lower anxiety scores, which supports the above results (Inanaga, 1998). It is also noteworthy that no severe or moderate adverse events occurred during the entire trial, with only two mild adverse events, both of which were post-treatment adverse reactions and resolved after a short rest period. This shows TBHM is safe for KOA patients.

This study offers initial insights into timing nocturnal melatonin-based interventions for KOA, but several limitations persist. First, this study primarily monitored circadian rhythms using salivary melatonin levels, lacking the measurement of other biomarkers such as inflammatory factors. And melatonin is only measured at the morning peak, lacking circadian curves. These may limit the interpretation of our results. Second, we used resting-state EEG data from patients before and after treatment, rather than monitoring EEG activity during sleep. This may have hindered the analysis of nocturnal cortical activity, hindering the understanding of a direct relationship between nocturnal pain and cortical activity. Furthermore, we did not stratify analyses by age or sex, which may have led to potential confounding factors that could affect our results. Future studies will investigate circadian rhythm-related biomarkers (such as interleukin-17) and monitor at multiple time points throughout the day and night, while monitoring KOA patients using sleep EEG. In addition, stratified analyzes will be also performed based on age, gender, and symptom severity to further validate the results of this study.

## 5 Conclusion

TBHM consistently outperforms joint mobilization in alleviating KOA pain, regulating cortical electrical activity, and boosting nighttime melatonin secretion. The therapeutic effect is enhanced when the intervention is closer to sleep time.

## Data Availability

The raw data supporting the conclusions of this article will be made available by the authors, without undue reservation.
